# Predictive Prognostic Model for Hepatocellular Carcinoma Based on Seven Genes Participating in Arachidonic Acid Metabolism

**DOI:** 10.1002/cam4.70284

**Published:** 2024-11-14

**Authors:** Xinyu Gu, Jing Wang, Jun Guan, Guojun Li, Xiao Ma, Yanli Ren, Shanshan Wu, Chao Chen, Haihong Zhu

**Affiliations:** ^1^ State Key Laboratory for Diagnosis and Treatment of Infectious Diseases, National Clinical Research Center for Infectious Diseases, National Medical Center for Infectious Diseases, Collaborative Innovation Center for Diagnosis and Treatment of Infectious Diseases, the First Affiliated Hospital Zhejiang University School of Medicine Hangzhou China; ^2^ Department of Hepatology The Second Hospital of Yinzhou of Ningbo Ningbo China; ^3^ Zhejiang University School of Medicine Hangzhou Zhejiang China

**Keywords:** arachidonic acid metabolism, hepatocellular carcinoma, immunotherapy responses, prognosis, tumor microenvironment

## Abstract

**Background:**

The occult onset and rapid progression of hepatocellular carcinoma (HCC) lead to an unsatisfactory overall survival (OS) rate. Established prognostic predictive models based on tumor‐node‐metastasis staging and predictive factors do not report satisfactory predictive efficacy. Arachidonic acid plays pivotal roles in biological processes including inflammation, regeneration, immune modulation, and tumorigenesis. We, therefore, constructed a prognostic predictive model based on seven genes linked to arachidonic acid metabolism, using samples of HCC patients from databases to analyze the genomic profiles. We also assessed the predictive stability of the constructed model.

**Methods:**

Sample data of 365 patients diagnosed with HCC were extracted from The Cancer Genome Atlas (TCGA, training set) and HCCDB18, GSE14520, and GSE76427 databases (validation sets). Patient samples were clustered using ConsensusClusterPlus analysis based on the expression levels of 12 genes involved in arachidonic acid metabolism that were significantly associated with HCC prognosis. Differentially expressed genes (DEGs) within different clusters were distinguished and compared using WebGestaltR. Immunohistochemistry (IHC) analysis was performed using a human HCC tissue microarray (TMA). Tumor immune microenvironment assessment was performed using ESTIMATE, ssGSEA, and TIDE.

**Results:**

Samples of patients with HCC were classified into three clusters, with significant differences in OS. Cluster 2 showed the best prognosis, whereas cluster 1 presented the worst. The three clusters showed significant differences in immune infiltration. We then performed Cox and LASSO regression analyses, which revealed CYP2C9, G6PD, CDC20, SPP1, PON1, TRNP1, and ADH4 as prognosis‐related hub genes, making it a simplified prognostic model. TMA analysis for the seven target genes showed similar results of regression analyses. The high‐risk group showed a significantly worse prognosis and reduced immunotherapy efficacy. Our model showed stable prognostic predictive efficacy.

**Conclusions:**

This seven‐gene–based model showed stable outcomes in predicting HCC prognosis as well as responses to immunotherapy.

**Abbreviations**
12‐HETE: 12‐hydroxyeicosatetraenoic acid
15‐HETE: 15‐hydroxyeicosatetraenoic acid
5‐LOX: 5‐lipoxygenase
ADH4: alcohol dehydrogenase 4
AUC: area under the curve
CDC20: cell division cycle 20 homolog
CDF: cumulative distribution function
CYP2C9: cytochrome P450 family 2 subfamily C member 9
DEGs: differentially expressed genes
EMT: epithelial‐mesenchymal transition
ESTIMATE: Estimation of STromal and Immune cells in Malignant Tumor tissues using Expression data
G6PD: glucose‐6‐phosphate dehydrogenase
GATK: Genome Analysis Toolkit
GEO: gene expression omnibus
GSEA: gene set enrichment analysis
HCC: hepatocellular carcinoma
HR: hazard ratio
ICB: immune checkpoint blockade
ICI: immune checkpoint inhibitor
KEGG: Kyoto Encyclopedia of Genes and Genomes
LASSO‐Cox: least absolute shrinkage and selection operator‐Cox
LIHC: TCGA‐liver HCC
lncRNA: long noncoding RNA
MEG3: maternally expressed gene 3
OIT_3_
: oncoprotein‐induced transcript 3
OS: overall survival
PCA: principal component analysis
PD/SD: progressive disease/stable disease
PGE_2_
: prostaglandins E_2_
PON1: paraoxonase 1
POR: cytochrome P450 oxidoreductase
ROC: receiver operating characteristic
SPP1: secreted phosphoprotein 1
ssGSEA: single‐sample gene set enrichment analysis
TCGA: The Cancer Genome Atlas
TIDE: Tumor Immune Dysfunction and Exclusion
TMB: tumor mutational burden
TME: tumor microenvironment
TNM: tumor‐node‐metastasis
TRNP1: TMF‐regulated nuclear protein 1

## 1 Introduction

Hepatocellular carcinoma (HCC), the fifth most common cancer and the third leading cause of cancer‐related deaths worldwide [1], imposes an increasing health and economic burden on patients in many countries [2]. The primary hazard factors for HCC encompass chronic infections of hepatitis B virus and hepatitis C virus, excessive alcohol consumption, diabetes mellitus, and, potentially, nonalcoholic fatty liver disease [3]. Despite advances in HCC treatment methods, such as resection of the pathological liver sections, radiotherapy, and liver transplantation [4, 5], the occult onset and rapid progression of HCC lead to an unsatisfactory rate of overall survival (OS), and patients with the late‐stage disease receive only palliative treatments [5], and the medium survival time of patients diagnosed with late‐stage HCC remains unsatisfactory [6]. Hence, strategies focusing on the prognostic prediction of patients diagnosed with HCC are always considered significant measures.

Arachidonic acid, also known as all‐cis‐5,8,11,14‐eicosatetraenoic acid, is an ω‐6 polyunsaturated fatty acid made of 20 carbon chains and four cis double bonds [7]. It serves as a crucial component of the biological cell membrane, vital for preserving its fluidity and flexibility [8]. Under the action of different enzymes, arachidonic acid is converted into diverse metabolic derivatives that play pivotal roles in biological processes such as inflammation [9], regeneration [10], adipogenesis [11], detoxification [7], coagulation [12], immune modulation [13], and tumorigenesis [14].

The tumor microenvironment (TME) consists of both tumor and nontumor cells such as immunocytes, stromal cells, and tumor‐related fibroblasts. The immunocytes within the TME play non‐negligible roles in the development of cancer [15]. As the tumor develops, the cells surrounding the tumor as well as soluble factors undergo alterations accordingly, which adds to the complexity of the TME [16]. In particular, immune cell infiltration in the TME substantially influences tumorigenesis and was actively enrolled in the inhibition and promotion of tumor cells [17, 18]. Thus, modulation of immune infiltration into the TME is currently an interesting anticancer strategy [19, 20].

Conventional strategies for guiding HCC treatment and predicting prognosis are generally dependent on the tumor‐node‐metastasis (TNM) classification system [21, 22]. Some studies have also assessed the efficacy of HCC scoring models when combining TNM staging with other predictive factors such as genes associated with ferroptosis [23] and immune cell infiltration [24]. However, the predictive efficacy of these scoring models has not been very satisfactory. Currently, novel predictive models of HCC prognosis have been developed based on genome sequencing technologies and bioinformatics analyses, such as prognostic models based on genes participating in cuproptosis [25], tumor immunological phenotypes [26], and m^6^A methyltransferase‐related long noncoding RNAs (lncRNAs) [27], based on clinical samples from The Cancer Genome Atlas (TCGA) and NCBI gene expression omnibus (GEO) databases, but only a few of these models have focused on predicting patients' responses to HCC immunotherapy.

Therefore, in our study, we applied Cox regression analysis and least absolute shrinkage and selection operator (LASSO)–Cox regression analysis for establishing a prognostic predictive model based on seven genes involved in arachidonic acid metabolism. To further test the predictive stability of the model, we assessed risk scores, time‐dependent receiver operating characteristic (ROC) curve, and Kaplan–Meier survival curves.

## 2 Methods

### 2.1 Data Collection and Processing

Data samples from 365 patients diagnosed with HCC were obtained from TCGA‐liver HCC (LIHC) dataset and were utilized as the training set to analyze mRNA expression levels as well as clinical follow‐up information. Samples with missing clinical data were excluded. The presence of somatic mutations containing single‐nucleotide variants was analyzed using the MuTect2 method by Genome Analysis Toolkit (GATK, v4.1.9.0). Similarly, gene data (*n* = 203) were retrieved from the HCCDB18 database (http://lifeome.net/database/hccdb/home.html), and gene data from GSE14520 (*n* = 221) and GSE76427 (*n* = 115) were acquired from the NCBI GEO database (https://www.ncbi.nlm.nih.gov/geo/). Additionally, immunotherapy datasets (IMvigor210, *n* = 298 [28]; GSE91061, *n* = 49; GSE78220, *n* = 27; GSE135222, *n* = 27) were obtained. These data were analyzed for validation of the model. In the present study, we collected patient data on age, sex, HCC grade, TNM stage, survival duration, and expression levels of associated genes.

The gene set enrichment analysis (GSEA) database (https://www.gsea‐msigdb.org/gsea/index.jsp) was used to obtain data on the arachidonic acid metabolism pathway and the genes involved in this pathway. A sum of 58 genes related to arachidonic acid metabolism were collected.

### 2.2 Identification of Arachidonic Acid Metabolism Subtypes

ConsensusClusterPlus software [29] of R packet was used for unsupervised consensus clustering of the TCGA‐LIHC and HCCDB18‐LIHC datasets based on the expression amplitude of 12 participating in arachidonic acid metabolism genes identified by univariate regression analysis. The cumulative distribution function (CDF) was used to evaluate clustering efficacy, and the optimal k value was determined through the evaluation of the delta area and the consensus matrix. Next, we conducted principal component analysis (PCA) on the 12 genes related to HCC prognosis for verification of the clustering results.

Furthermore, the immune infiltration condition of each subtype was assessed by single‐sample gene set enrichment analysis (ssGSEA), based on the enrichment levels of immune signatures, including the infiltration abundance of 28 immunocytes [30] and 27 immune reaction activities [31] reported in published studies.

### 2.3 Construction of the HCC Prognostic Model Based on Genes Related to Arachidonic Acid Metabolism

For the HCC subtypes, a pairwise comparison was performed using the “Limma” package in R (|log2 (fold change)| > 1, false discovery rate–adjusted *p* < 0.05) to identify differentially expressed genes (DEGs) [32]. Next, a comparison of C1/C2, C1/C3, and C2/C3 was performed in pairs to reveal three groups of DEGs, and the results were displayed as a Venn diagram.

Next, the overlapping DEGs were assessed in univariate Cox regression analysis, and DEGs significantly affecting HCC prognosis were identified (*p* < 0.01). Then, we performed LASSO‐Cox regression analysis using the “glmnet” function of the R package [33], which further filtered these genes and revealed the seven hub genes to establish the predictive model. A final prognostic signature of the genes participating in arachidonic acid metabolism was acquired by summing the LASSO‐Cox regression model coefficients (*β* values) multiplied by the corresponding mRNA levels.

### 2.4 Validation of the Performance and Prognostic Capability of the Seven‐Gene–Based Model

After construction of the HCC model, patient samples were categorized into two groups, namely, high‐ and low‐risk groups, with the optimal cutoff risk score determined using the “surv_cutpoint” algorithm for survival analysis in the R package [34]. A Kaplan–Meier survival analysis was then utilized for evaluating significant differences in OS between both groups. ROC curves were plotted using the “timeROC” algorithm of the R package [35], and the area under the curve (AUC) values for 1, 3, and 5 years were calculated to evaluate the prognostic efficacy of the model. Next, the log‐rank test was used to determine significant differences in terms of survival rate. The correlation between risk score and clinical parameters such as T stage, N stage, M stage, tumor stage, and grade was analyzed using variance analysis. The score for the Kyoto Encyclopedia of Genes and Genomes (KEGG) arachidonic acid metabolism pathway was calculated using the ssGSEA method included in the R package “GSVA” [36] and analyzed for its correlation with the clinical parameters.

### 2.5 Exploration of the Gene Mutation Landscape

The TCGA‐LIHC mutation data were processed using mutect2 software, resulting in the identification of 2564 genes with a mutation frequency exceeding 3. Fisher's exact test was then employed to discern genes exhibiting a significantly elevated mutation frequency (*p* < 0.05) in both the high‐ and low‐risk groups. A heatmap was then generated, depicting the top 20 mutated genes within these risk groups. Next, the correlations of tumor mutational burden (TMB) and risk type with patient OS were analyzed.

### 2.6 Evaluation of the Seven‐Gene–Based Model Using Tissue Microarray Technology

The tissue microarray (TMA, HLivH180Su30, purchased from Shanghai Outdo Biotech Co. Ltd) contained human HCC tissues along with adjacent nontumor tissues collected from 90 patients. Prior to the procurement of these tissues, informed consent was obtained from all participating patients, ensuing ethical compliance and respecting their rights. Primary antibodies against CYP2C9 (ab4236; Abcam, Cambridge, MA, USA), G6PD (EPR20668, ab210702; Abcam), CDC20 (CDC20/1102, ab215908; Abcam), SPP1 (YT3467; ImmunoWay, Plano, TX, USA), PON1 (EPR2892, ab92466; Abcam), TRNP1 (EPR11958, ab174303; Abcam), and ADH4 (EPR9483, ab137077; Abcam) were added to the IHC‐stained tissues, subsequently followed by anti‐mouse/anti‐rabbit secondary antibodies incubation.

For each sample, the IHC staining score was evaluated based on two criteria: intensity of staining and percentage of tumor cells stained. Intensity of staining was categorized into four levels from level 0 to 3, standing for staining gradient including zero, weak, moderate, and strong staining. The percentage of stained tumor cells was also categorized into four groups: level 1 (0% ≤ × ≤ 25%), level 2 (25% < × ≤ 50%), level 3 (50% < × ≤ 75%), and level 4 (75% < × ≤ 100%). Final staining scores of each sample were determined by multiplying the two variables, which ranged from 0 to 12. Furthermore, all samples were categorized into the low‐ and high‐score groups (< 4 and ≥ 4, respectively). For each gene among the seven genes, a Kaplan–Meier survival analysis was performed to assess the significant difference in OS between the two groups.

### 2.7 Assessment of the Predictive Ability of the Model for Immunotherapy Efficacy

To explore the effectiveness of our model in predicting the immunotherapy outcome, the high‐ and low‐risk groups from the TCGA‐LIHC cohort were compared with regards to differences in infiltration of immunocytes, immunal reaction activities, immune checkpoint‐related gene expression levels, as well as immunotherapy responses. The ssGSEA algorithm [36] was utilized to quantify the relative abundance of infiltrating immunocytes and immune reaction activities. The Estimation of Stromal and Immune cells in Malignant Tumor tissues using Expression data (ESTIMATE) algorithm was used to assess the immune scores of TCGA‐LIHC samples. Next, immune checkpoint‐related gene expression levels were analyzed, as they potentially reflect treatment responses during immune checkpoint blockade (ICB). The 13 immune checkpoint‐related genes, identified from previously published studies, were selected, and the ssGSEA algorithm was utilized to compute the immune scores of both groups. The Tumor Immune Dysfunction and Exclusion (TIDE, http://tide.dfci.harvard.edu/) algorithm was also used for estimation of clinical immunotherapy responses in both risk groups in TCGA‐LIHC samples. A higher TIDE score usually reflected an enhanced possibility of immune escape and indicated a lower possibility that patients could benefit from immunotherapy. Furthermore, the pRRophetic algorithm of the R package [37] was used to predict tumor sensitivity to traditional chemotherapy in both high‐ and low‐risk groups.

Finally, immunotherapy datasets (IMvigor210, GSE91061, GSE78220, and GSE135222) were analyzed using our predictive model, and the results were validated in Kaplan–Meier survival analysis.

### 2.8 Statistical Analysis

Statistical analyses were conducted using SPSS software v25 (IBM, Chicago, IL, USA), GraphPad Prism 7.0 (GraphPad Software, La Jolla, CA, USA), and R software (version 3.5.1). Intergroup statistical comparisons were performed by Student's t‐test, and the Kruskal–Wallis test was performed in conditions of multiple comparisons. The Kaplan–Meier method was adopted to analyze patients' OS, followed by the log‐rank test. A *p* < 0.05 was considered statistically significant.

## 3 Results

### 3.1 Landscape of Genes Related to Arachidonic Acid Metabolism in HCC

Overall, 58 genes participating in arachidonic acid metabolism were identified from the GSEA website (http://www.gsea‐msigdb.org/). Then, we performed univariate Cox regression analysis to identify survival‐related genes in the TCGA‐LIHC training dataset, and the results revealed 12 genes participating in arachidonic acid metabolism (GPX4, GGT7, PTGES2, AKR1C3, PTGS1, HPGDS, TBXAS1, GPX7, PTGES, CYP2C8, CYP4A11, and CYP2C9) as potential prognostic indicators of OS of patients with HCC, nine of which were risk factors and three were protective (Figure S1A). Next, we analyzed the differences in the expression levels of the 12 genes related to prognosis in cancerous and paracancerous tissues, six of which showed significantly upregulated expression in cancerous tissues and five showed significantly downregulated expression levels (Figure S1B).

Moreover, we assessed the mutation frequency of the 12 genes participating in arachidonic acid metabolism in HCC, suggesting that these genes rarely had mutations. The mutation frequency in CYP2C8, CYP2C9, GGT7, PTGS1, and CYP4A11 was 1%, and that in the other genes was less than 1% (Figure S1C). Finally, the copy number variation for the 12 genes participating in arachidonic acid metabolism in HCC was examined and visualized (Figure S1D).

### 3.2 Description of HCC Subtypes Based on Arachidonic Acid Metabolism

**FIGURE 1:**
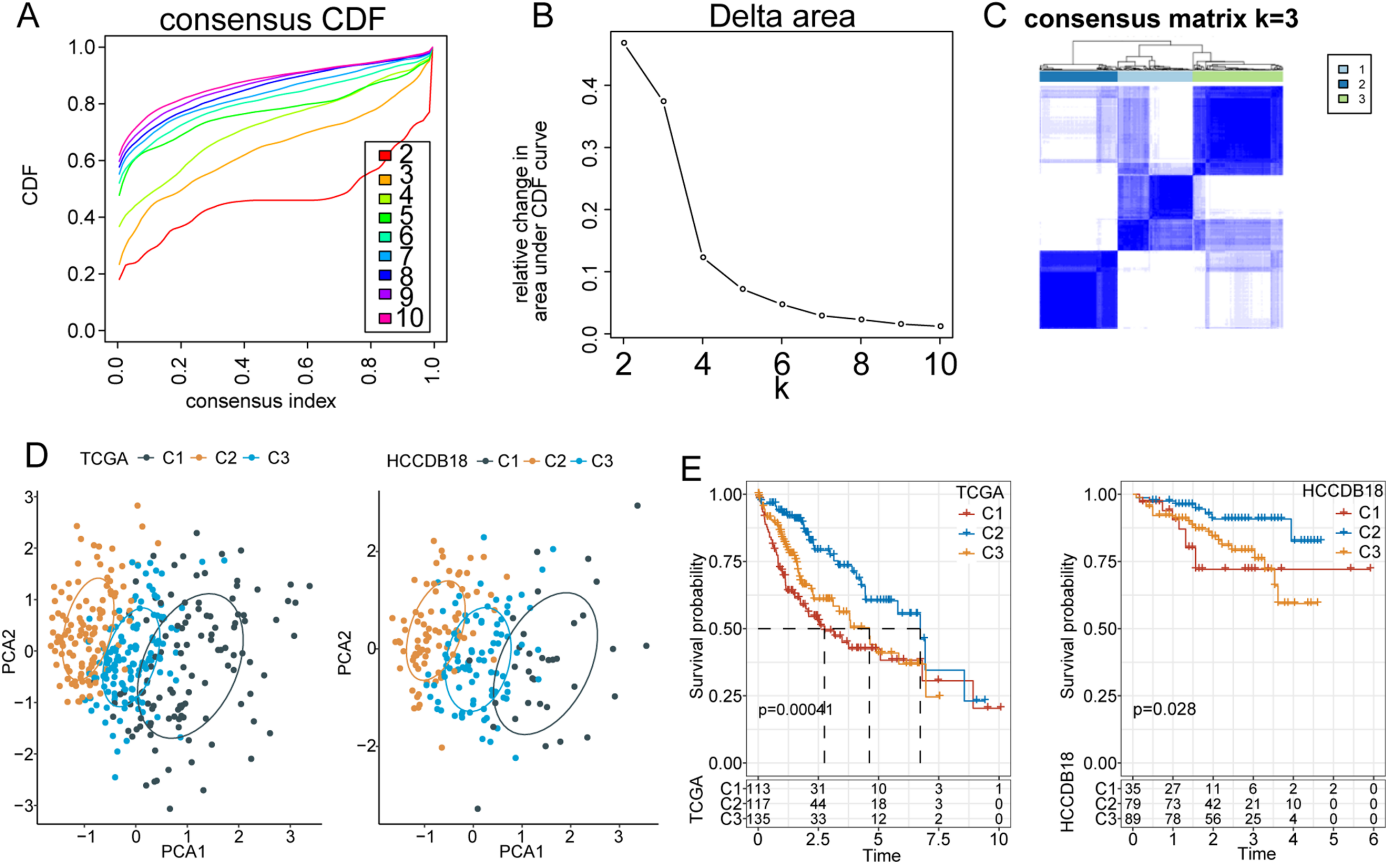
Analysis of HCC subtypes based on arachidonic acid metabolism. (A–C) Consensus clustering results of TCGA‐LIHC samples. (D) Principal component analysis distribution demonstrating clear boundaries for the three HCC subtypes in both TCGA‐LIHC and HCCDB18‐LIHC samples. (E) Kaplan–Meier survival analysis showing significant differences in OS for each subtype. HCC: hepatocellular carcinoma; LIHC: liver hepatocellular carcinoma; OS: overall survival; TCGA: The Cancer Genome Atlas.

Consensus clustering was performed on TCGA‐LIHC samples to separate the HCC samples into subtypes based on the mRNA expression levels of the 12 genes participating in arachidonic acid metabolism in HCC. CDF was used to evaluate the clustering efficacy. The optimal value of k was determined to be 3, where the relative change in AUC reached an approximate maximum. Concurrently, the consensus matrix displayed a distinct boundary, indicating a stable and robust clustering pattern (Figure 1A–C). Therefore, three distinguished HCC subtypes were classified, designated as C1, C2, and C3. Next, PCA was performed based on the 12 genes participating in arachidonic acid metabolism in HCC for the verification of the clustering results. The principal component distribution demonstrated clear boundaries for the three HCC subtypes in both TCGA‐LIHC and HCCDB18‐LIHC samples, consistent with the consensus matrix results, which confirmed the stability and reliability of consensus clustering analysis (Figure 1D).

**FIGURE 2:**
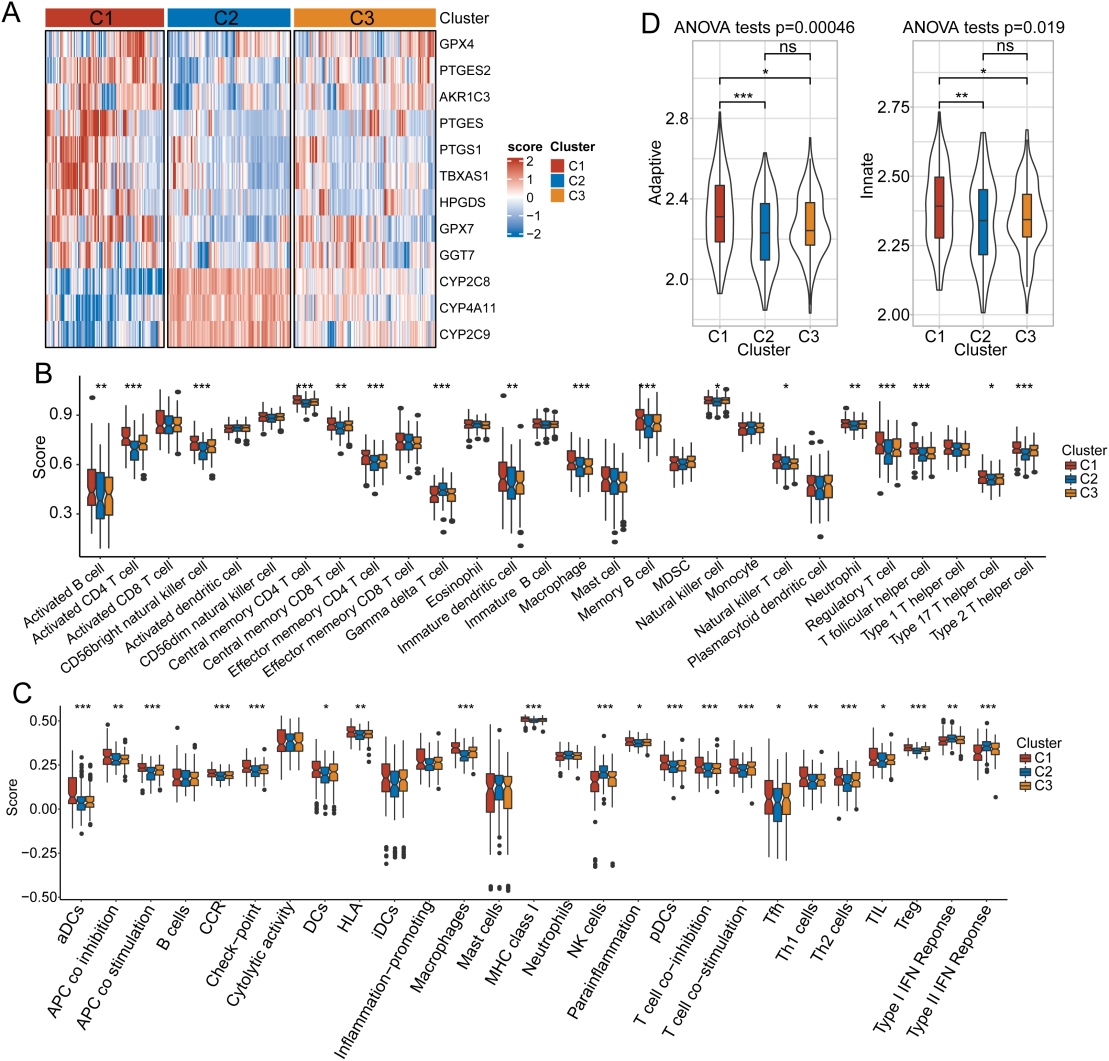
Analysis of the expression of prognosis‐related genes and immune conditions of HCC subtypes. (A) Heatmap showing the expression levels of 12 prognosis‐related genes in each TCGA‐LIHC subtype. (B, C) Immune cell infiltration condition in each subtype. (D) Analyses of acquired and innate immune responses in each subtype. HCC: hepatocellular carcinoma; LIHC: liver hepatocellular carcinoma; TCGA: The Cancer Genome Atlas. *p < 0.05, **p < 0.01, ***p < 0.001.

Additionally, a Kaplan–Meier survival analysis was conducted for each TCGA‐LIHC and HCCDB18‐LIHC subtype, which showed significant differences in OS for each subtype; the C2 subtype had the best prognosis while the C1 subtype showed the worst (Figure 1E). For the C3 subtype, a medium level of patient prognosis was shown (Figure 1E). A heat map showing the mRNA expression levels of the 12 genes participating in arachidonic acid metabolism in the three TCGA‐LIHC subtypes was constructed (Figure 2A). The C2 subtype generally featured the highest gene expression level, while the C1 subtype had the lowest.

Furthermore, the immune cell infiltration in each subtype was analyzed using ssGSEA based on the enrichment levels of immune signatures, including the abundance of infiltration of 28 immunocytes [30] (Figure 2B) and 27 immune reaction activities [31] (Figure 2C) that were reported in published studies. The results displayed the highest immune score for the C1 subtype and the lowest score for the C2 subtype, indicating a positive relationship between enhanced immune cell infiltration and adverse prognostic outcomes. Analyses of acquired and innate immune responses showed similar results (Figure 2D). Collectively, the findings confirmed the presence of heterogeneity in arachidonic acid metabolism in patients with HCC, which exerted pivotal impacts on the prognosis of HCC.

### 3.3 Construction of the Seven‐Gene–Based HCC Prognostic Model

**FIGURE 3:**
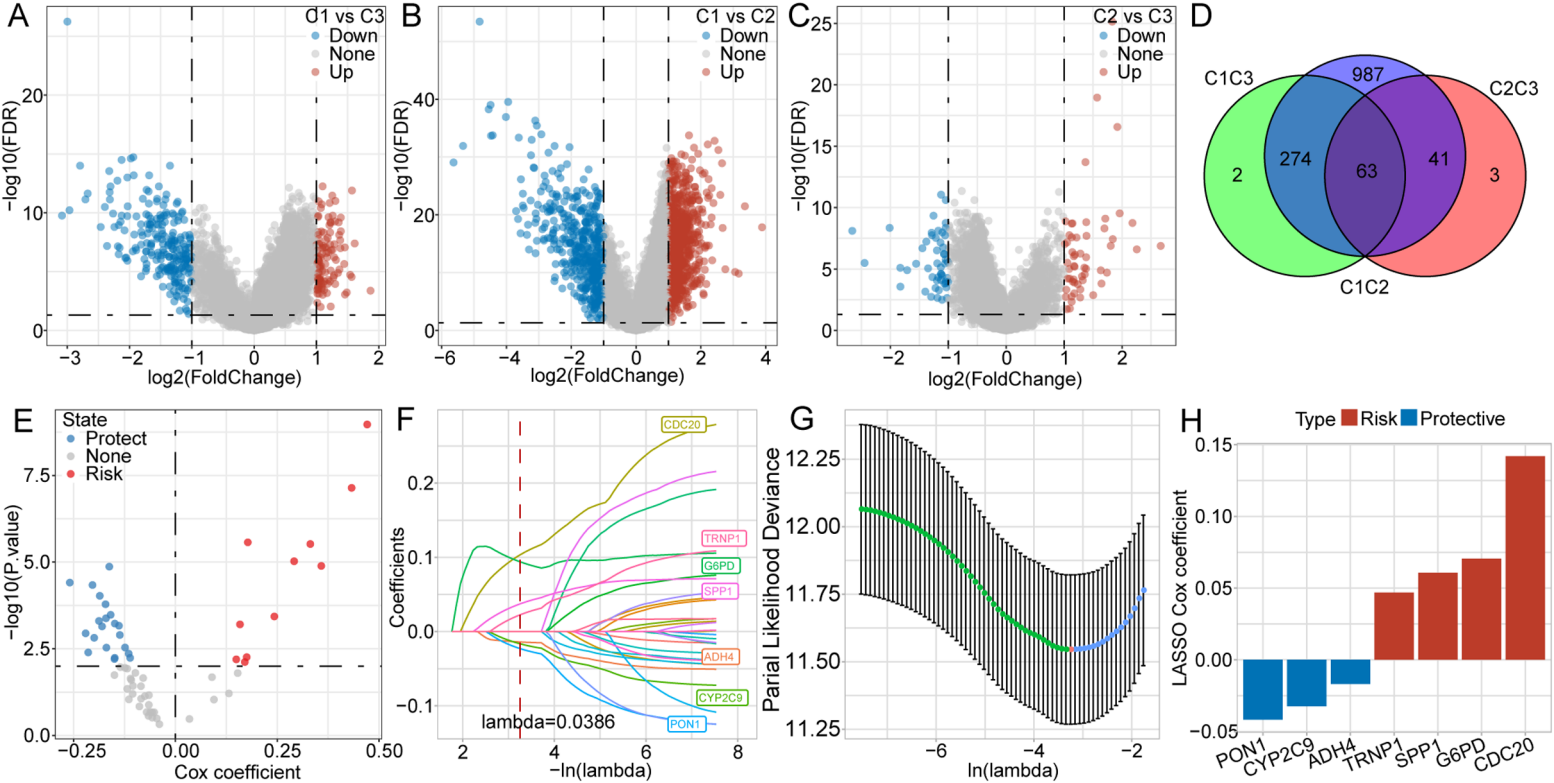
Construction of the seven‐gene–based HCC prognostic model. (A–D) DEGs among subtypes pairwise. A total of 63 common DEGs were identified. (E) A total of 32 common DEGs significantly associated with HCC prognosis, 11 of which were risk factors while 21 were protective factors. (F–H) LASSO regression model used for compressing the genes in the HCC prognostic model. Seven genes (CYP2C9, G6PD, CDC20, SPP1, PON1, TRNP1, and ADH4) were selected as hub genes. DEG: differentially expressed gene; HCC: hepatocellular carcinoma; LASSO: least absolute shrinkage and selection operator.

DEGs among the three HCC subtypes were obtained pairwise using the limma package (Figure 3A–C). KEGG pathway and GO functional enrichment analyses were performed using the WebGestaltR package (v0.4.4). Next, the three groups of DEGs were intersected, which revealed 63 common DEGs (Figure 3D). We then applied univariate Cox regression analysis on the common DEGs, which revealed 32 genes significantly associated with prognosis in HCC (*p* < 0.01), 11 of which were risk factors while 21 were protective factors (Figure 3E). The LASSO regression model was then used to further compress these 32 genes based on TCGA‐LIHC samples to compress the number of hub genes in the HCC prognostic model. As the lambda value gradually increased, the number of related independent variable coefficients tending toward zero increases as well (Figure 3F). Ten‐fold cross‐validation was performed during the construction of the model. Confidence intervals showed that the optimal model had a lambda value of 0.0386 (Figure 3G). Thus, seven genes (CYP2C9, G6PD, CDC20, SPP1, PON1, TRNP1, and ADH4) were identified as hub target genes for subsequent analyses. Finally, multivariate Cox regression analysis was performed to obtain the risk coefficient of each gene. The risk score was acquired by adding together the mRNA expression level of each of the seven genes multiplied by the corresponding LASSO coefficients. Risk score = −0.032 × CYP2C9 + 0.071 × G6PD + 0.142 × CDC20 + 0.061 × SPP1–0.042 × PON1 + 0.047 × TRNP1–0.017 × ADH4. Among the seven genes, CDC20, G6PD, SPP1, and TRNP1 had positive coefficients, while PON1, CYP2C9, and ADN4 showed a contrasting result (Figure 3H). The seven‐gene–based model was then further evaluated for its dependability in the following steps.

### 3.4 Evaluation and Validation of the Predictive Efficacy of the Seven‐Gene–Based Model

**FIGURE 4:**
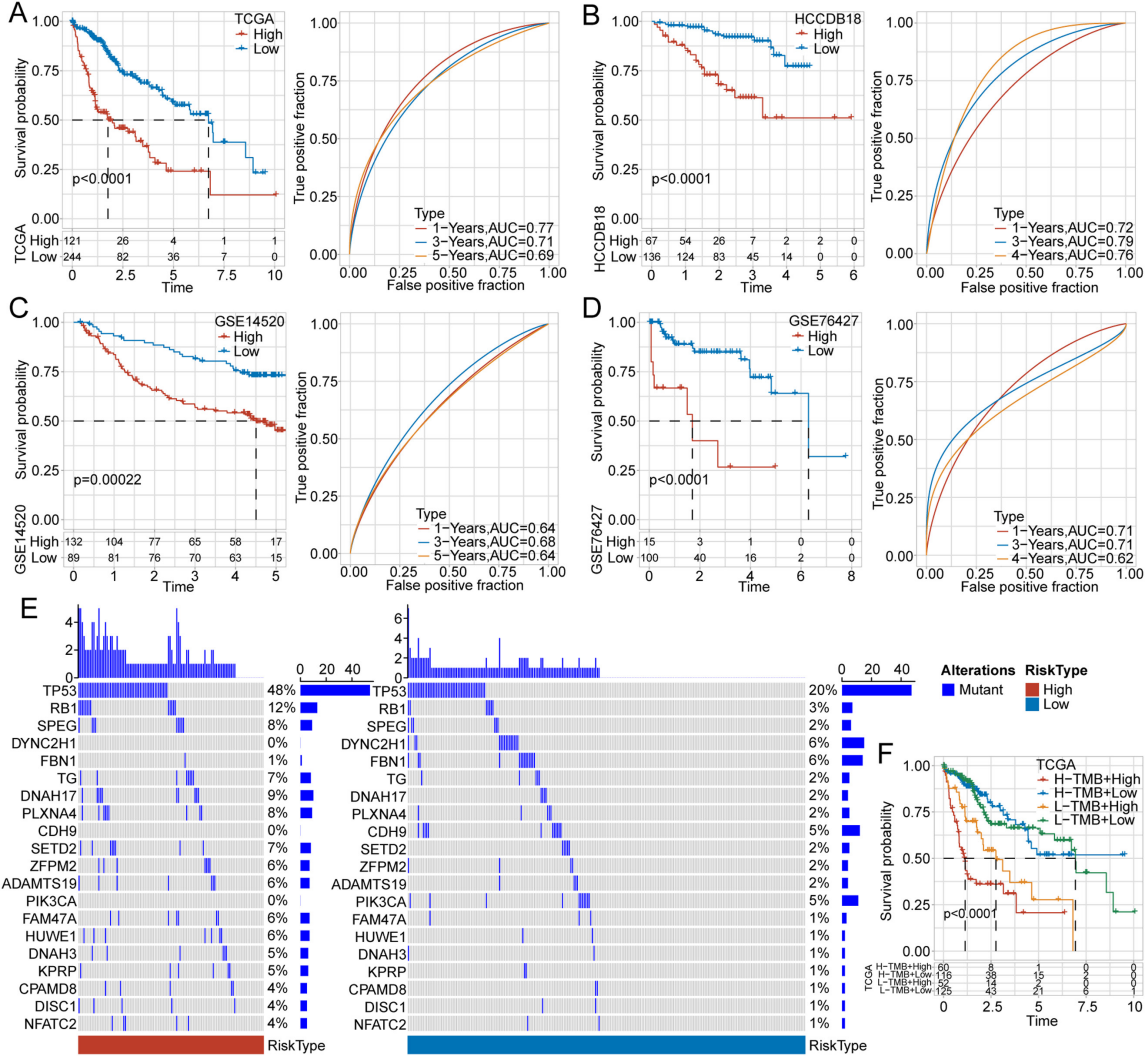
Evaluation and validation of the predictive efficacy of the seven‐gene–based model, and correlation between risk score and gene mutation. (A–D) Kaplan–Meier survival analysis results confirmed significant differences in the OS of patients with HCC in the TCGA‐LIHC training dataset and HCCDB18, GSE14520, and GSE76427 validation datasets. Time‐dependent ROC curve (timeROC) showing satisfactory prediction accuracy of the model on the training and validation datasets. (E) Top 20 mutated genes in both the high‐ and low‐risk groups. (F) Risk group and TMB levels having significant effects on patient OS. HCC: hepatocellular carcinoma; LIHC: liver hepatocellular carcinoma; OS: overall survival; ROC: receiver operating characteristic; TCGA: The Cancer Genome Atlas; TMB: tumor mutational burden.

To further evaluate the association between the risk score based on the model and survival outcomes of HCC, we determined the optimal cutoff value for risk scores using the surv_cutpoint function in the R package survminer, and we separated the patient samples into the high‐ and low‐risk groups. Kaplan–Meier survival analysis was then employed on the TCGA‐LIHC training dataset, which suggested a significant difference in the patients' OS for at least 5 years (*p* < 0.0001; Figure 4A). Patients with higher risk scores displayed worse OS in the TCGA‐LIHC dataset. This OS divergence remained consistent in the HCCDB18 (*p* < 0.0001; Figure 4B), GSE14520 (*p* = 0.00022; Figure 4C), and GSE76427 (*p* < 0.0001; Figure 4D) validation datasets. Additionally, we employed the time‐dependent ROC curve (timeROC) method to estimate prediction accuracy using the TCGA‐LIHC training dataset as well as the HCCDB18, GSE14520, and GSE76427 validation datasets. By calculating the AUC values of 1, 3, and 5 years, we obtained satisfactory outcomes, indicating the robustness and reliability of the prediction model (Figure 4A–D).

### 3.5 Correlation Analysis Between Risk Score and Gene Mutation and Tumor Mutation Burden

We assessed genomic alterations in the TCGA‐LIHC samples in both risk groups, and 57 genes had a significantly higher mutation frequency (*p* < 0.05). The top 20 mutated genes in both groups are shown in Figure 4E.

Furthermore, we intersected the risk group and TMB and performed Kaplan–Meier survival analysis, and the results demonstrated a significant effect of risk group and TMB on patient OS (Figure 4F), indicating that high TMB levels were one of the causes for patients' worse prognosis.

### 3.6 Clinical Pathological Characteristics of Patients With Different Risk Scores

**FIGURE 5:**
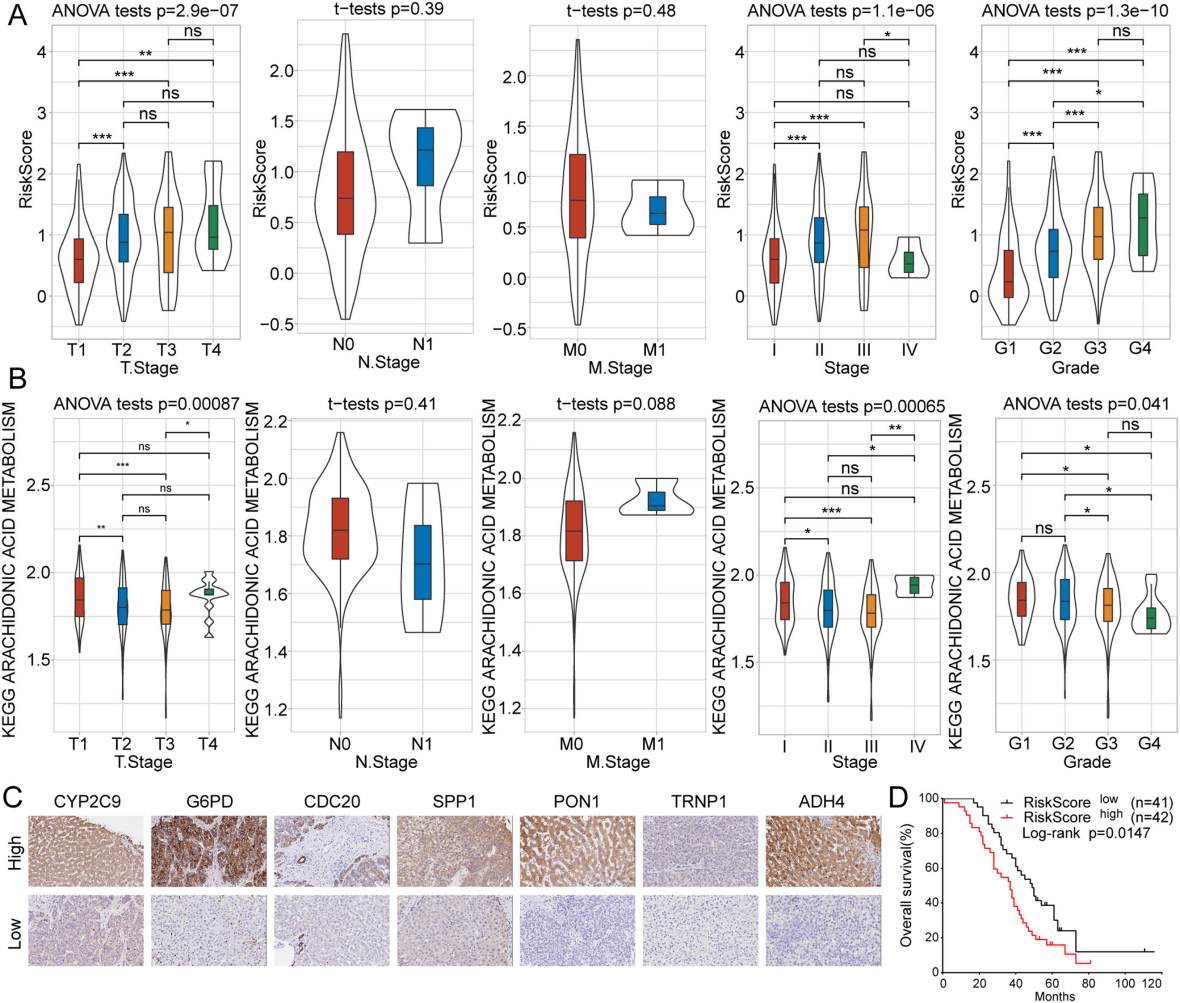
Clinical pathological characteristics of patients with different risk scores, and the results of TMA analysis, and Kaplan–Meier survival analysis based on clinical HCC samples. (A) Patients with different risk scores having significantly different T stage and tumor stage and grade. (B) Patients in different Kyoto Encyclopedia of Genes and Genomes arachidonic acid metabolism pathway score groups having significantly different T stage and tumor stage and grade. (C) IHC images showing the expression of seven proteins in HCC tumor tissues and adjacent normal tissues in TMA analysis. (D) Kaplan–Meier survival analysis indicating that patients with lower risk scores had better outcomes than those with higher risk scores. HCC: hepatocellular carcinoma; IHC: immunohistochemistry; TMA: tissue microarray. *p < 0.05, **p < 0.01, ***p < 0.001.

We investigated clinical pathological characteristics including T stage, N stage, M stage, and tumor stage and grade using TCGA‐LIHC samples, and the results showed significant differences in risk scores in patients whose T stages and tumor stages and grades differed (Figure 5A). Subsequently, scores for KEGG pathways related to arachidonic acid metabolism were also analyzed, and the results suggested significant differences in KEGG scores for the arachidonic acid metabolism pathway in patients with different T stages and tumor stage and grade (Figure 5B). These findings collectively indicated that our seven‐gene–based model was at least partly helpful for HCC tumor staging.

### 3.7 Tissue Microarray Analysis Results Further Validate the Seven‐Gene–Based Model

To further evaluate the model's efficacy and reliability, we performed TMA analysis to assess the protein expression level of the seven genes in samples of HCC tissues and adjacent nontumor tissues from 90 patients with HCC. Each sample was categorized as having high or low expression based on the calculated IHC staining score, the representative images of which are illustrated in Figure 5C. As expected, protein expression of CYP2C9, PON1, and ADH4 had dropped in the HCC tissues when compared with that in the adjacent normal tissues, while that of G6PD, CDC20, SPP1, and TRNP1 had increased. Additionally, statistical analysis revealed that patients whose tissues featured higher expression levels of CYP2C9 (*p* < 0.001), PON1 (*p* = 0.003), and ADH4 (*p* < 0.001) derive significantly better clinical outcomes, whereas those whose tissues have higher expression levels of G6PD (*p* = 0.002), CDC20 (*p* < 0.001), SPP1 (*p* < 0.001), and TRNP1 (*p* = 0.013) had a worse disease state. In addition, Kaplan–Meier survival analysis revealed that patients with lower risk scores had improved outcomes than those with higher risk scores (Figure 5D). Taken together, these results further confirmed the efficacy of our seven‐gene–based model.

### 3.8 Assessment of the Predictability of the Seven‐Gene–Based Model for Immunotherapy Efficacy

The results of ssGSEA revealed 40 pathways that had significant differences between both risk groups. Pathways related to cellular metabolism, such as oxidative phosphorylation, peroxisome, bile acid metabolism, xenobiotic metabolism, and fatty acid metabolism, were significantly upregulated in the high‐risk group, whereas pathways associated with cell cycle, such as DNA repair, MYC targets, E2F targets, G2/M checkpoint, and mitotic spindle, were significantly downregulated (Figure S2A).

Next, the immune infiltration condition of both groups was analyzed using ssGSEA based on the enrichment levels of immune signatures, including the abundance of the infiltration of 28 immunocytes [30] (Figure S2B) and 27 immune reaction activities [31] (Figure S2D) as reported in published studies. We discovered a correlation between higher immune scores and a higher risk of poor prognosis, and the results were similar in both acquired and innate immune responses (Figure S2C). Furthermore, the ESTIMATE algorithm was utilized to assess the immune scores of the TCGA‐LIHC samples, and the results showed a significantly higher immune score (*p* = 0.0013) in the high‐risk group than in the low‐risk group (Figure S2E). These results collectively indicated a relationship between immune cell infiltration and a high risk of poor prognosis.

We next investigated 13 groups of genes of interest in humans, as reported in a published study, using ssGSEA and studied the association between risk score and the expression levels of these genes. The results revealed a significant positive relevance between risk score and the expression of genes related to FGFR3, EMT1‐3, base excision repair, nucleotide excision repair, homologous recombination, mismatch repair, DNA replication, DDR, and cell cycle (Figure S2F).

Furthermore, the TIDE algorithm was used for the estimation of potential clinical immunotherapy response in both risk groups in TCGA‐LIHC samples. The low‐risk group had lower TIDE scores, indicating patients in the low‐risk group had a higher likelihood to derive benefits from immunotherapy (Figure S2G). We further evaluated the association between the expression level of each of the seven genes and important immunotherapy‐related features including T‐cell dysfunction, ICB immunotherapy response, phenotypes in CRISPER screens, and immunosuppressive cell types and visualized the results as a heatmap (Figure S2H). In addition, the pRRophetic algorithm was used to predict tumor sensitivity to traditional chemotherapy in both risk groups. Among the 72 drugs investigated, 65 were more effective in treating patients of the high‐risk group, whereas seven were found otherwise (Figure S2I).

**FIGURE 6:**
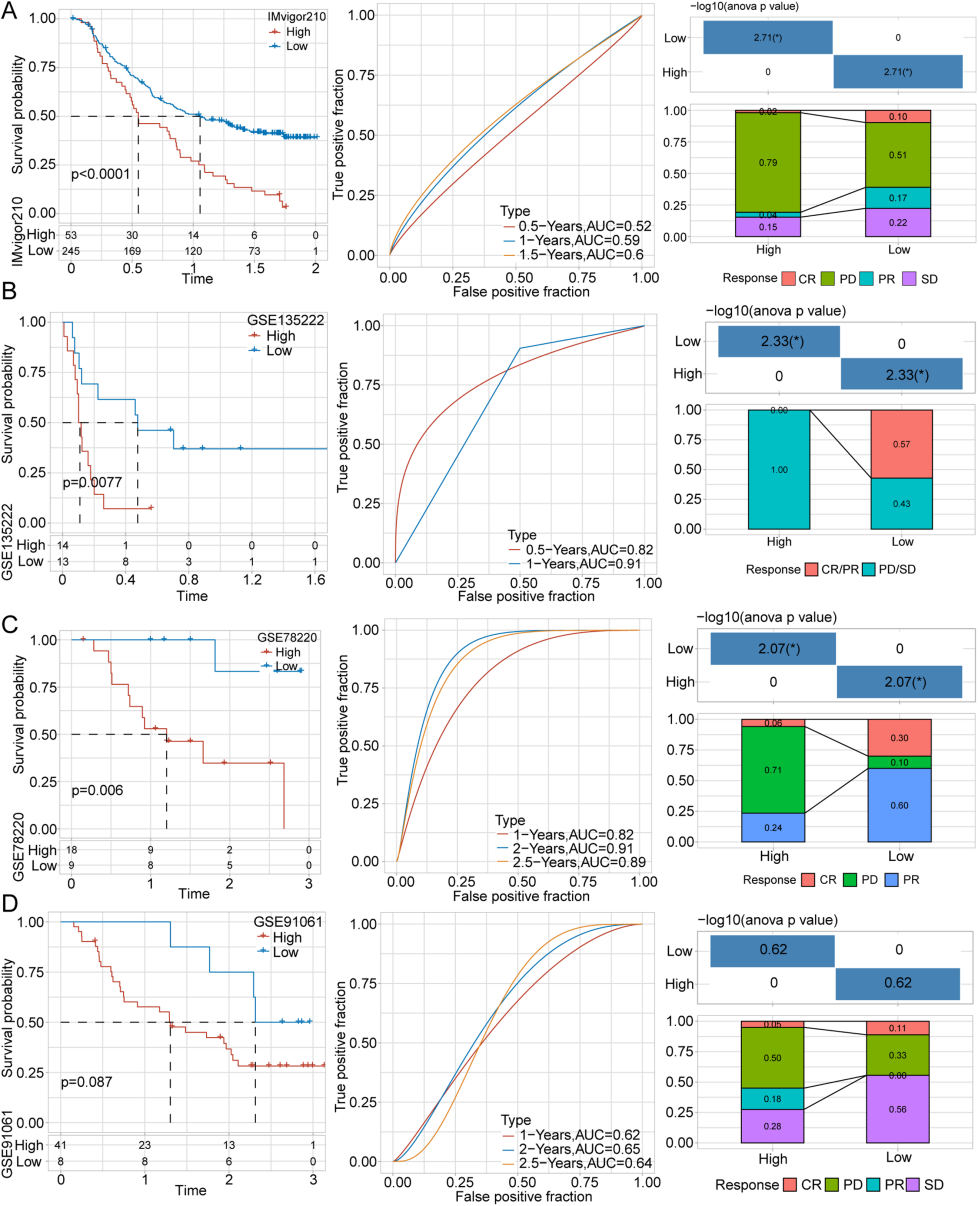
Results of Kaplan–Meier survival analysis based on samples of patients with HCC who received immunotherapy. (A–D) High‐ and low‐risk groups having significant difference in OS in the IMvigor210, GSE135222, and GSE78220 samples but not in the GSE91061 samples. The PD/SD ratio in the high‐risk group was higher than that in the low‐risk group, indicating a lower likelihood of patients within the high‐risk group to benefit from immunotherapy. HCC: hepatocellular carcinoma; OS: overall survival. *p < 0.05.

Finally, to assess tissue samples of patients who received immunotherapy, data were extracted from the IMvigor210, GSE91061, GSE78220, and GSE135222 databases. For each dataset, we numerated the risk score for each data sample, determined the optimal cutoff value, constructed curves of Kaplan–Meier survival analysis, and analyzed the PD/SD (progressive disease/stable disease) ratio. As shown in Figure 6A–D, both risk groups had significant differences in IMvigor210 (*p* < 0.0001), GSE135222 (*p* = 0.0077), and GSE78220 (*p* = 0.006) samples but not in the GSE91061 samples (*p* = 0.087). Moreover, the PD/SD ratio in the high‐risk group was higher than that in the low‐risk group, indicating a lower likelihood of patients in the high‐risk group benefiting from immunotherapy, a finding consistent with the conclusion of published studies.

Taken together, these results provide valuable information for the treatment of patients with HCC.

### 3.9 Seven‐Gene–Based Model Serves as an Independent Predictive Tool in Clinical Applications

**FIGURE 7:**
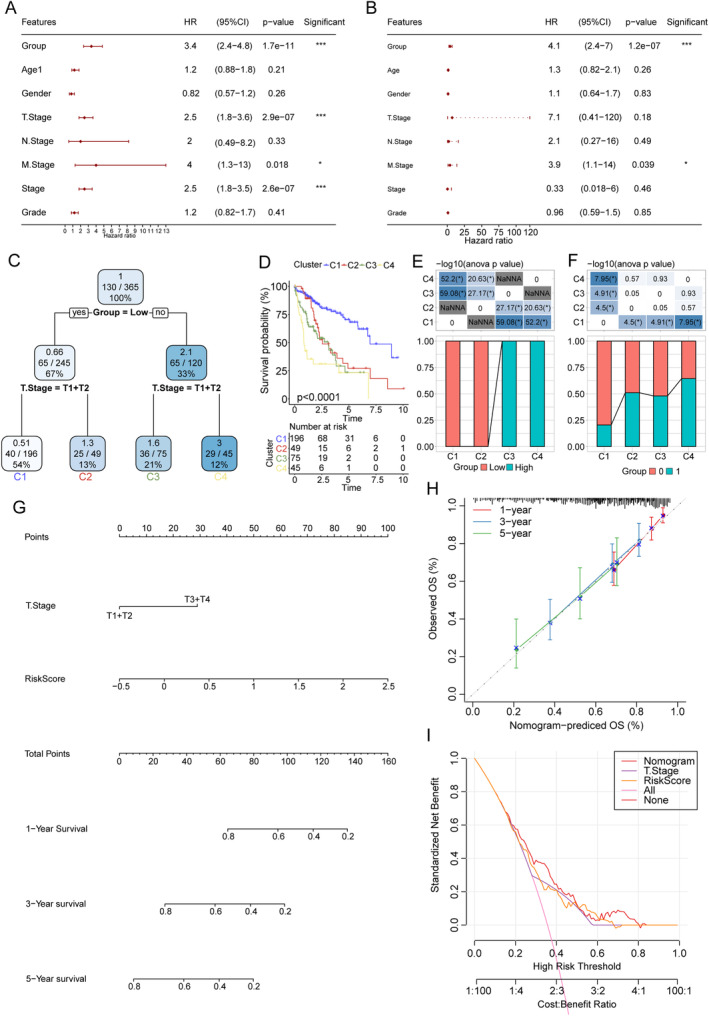
Risk score combined with clinical pathological characteristics further improves the HCC prognostic model. (A, B) Univariate and multivariate Cox analyses of RiskScore and clinicopathological features. (C) A decision tree constructed using risk group and T stage as pivotal parameters categorizing the samples into four different subtypes. (D–F) Samples from each subtype displaying significant differences in survival analysis. (G) A nomogram constructed based on risk scores and other clinicopathological features revealing risk score as the most important prognostic factor for HCC prognosis. (H) Calibration curve for the analysis of the model's accuracy. (I) DCA to assess the reliability of the prognostic model. DCA: decision curve analysis; HCC: hepatocellular carcinoma. *p < 0.05.

We assessed the potential of the seven‐gene–based model as an independent predictive tool in clinical practices. Univariate and multivariate Cox regression analyses of risk score and clinicopathological parameters including age, sex, T stage, M stage, N stage, and tumor stage and grade of TCGA‐LIHC samples were performed, and the results indicated that T stage (hazard ratio [HR] = 7.1) and risk group (HR = 4.1) had the highest HR among all parameters (Figure 7A,B). However, multivariate Cox regression analysis indicated only risk group (*p* = 1.2e‐07) and M stage (*p* = 0.039) as independent biomarkers of statistical significance (Figure 7B). These results confirmed that our seven‐gene–based model served as an independent predictive tool in HCC prognosis prediction.

### 3.10 Risk Score Combined With Clinical Pathological Characteristics Further Improves the HCC Prognostic Model

We then created a decision tree based on risk group and clinicopathological parameters including age, sex, T stage, M stage, N stage, and tumor stage and grade of samples from the TCGA‐LIHC cohort. The risk group and T stage were determined as pivotal parameters and were used to categorize the samples into four different subtypes (Figure 7C). Samples from each subtype displayed significant differences in survival analysis (Figure 7D–F), indicating the predictive prognostic value for HCC in the clustering based on risk group and T stage.

To further quantify the prognostic risk and survival expectation of each patient with HCC, we established a nomogram based on risk scores and other clinicopathological features (Figure 7G), which revealed the risk score as the most important prognostic factor. Moreover, calibration curves were constructed to assess the model's prognostic accuracy. In general, the calibration plots suggested an excellent calibration agreement of observed probabilities with predicted probabilities of HCC samples at 1 year, 3 years, and 5 years, ensuring the clinical prognostic implications of the model (Figure 7H). Finally, decision curve analysis was performed to assess the dependability of the prognostic model. As illustrated in Figure 7I, a comparison of the net benefits of using different prognostic predictive strategies was performed for stratifying people, including our seven‐gene–based model, T stage, and the nomogram combining the two factors, and the extreme strategies of screening every one or no one, in all the TCGA‐LIHC samples investigated (Figure 7I). The results showed that our seven‐gene–based model would have an improved prognosis prediction value when combined with clinical pathological characteristics.

## 4 Discussion

Arachidonic acid plays a pivotal role in the progression and inhibition of HCC; however, there still exists a lack of comprehensive knowledge of the underlying mechanisms. For example, as reported by Yong‐Jiang Xu et al., eicosanoids derived from arachidonic acid, including PGE_2_ (prostaglandins E_2_), 5‐LOX (5‐lipoxygenase), 12‐HETE (12‐hydroxyeicosatetraenoic acid), and 15‐HETE, elevated hepatic inflammation as well as liver regeneration, thus favoring HCC progression [38]. Wen et al. reported that the liver‐specific tumor‐suppressive OIT_3_ (oncoprotein‐induced transcript 3) increased ferroptosis in patients with HCC partly due to the stimulation of arachidonic acid metabolism [39]. In our study, we determined that four of the seven prognosis‐related genes identified in LASSO‐Cox regression analyses revealed a positive correlation with risk score, whereas the remaining three showed a contrasting result. This result further supported the complex and dual functions of arachidonic acid metabolism in the context of HCC.

Among the seven genes in the model, four (G6PD, CDC20, SPP1, and TRNP1) were positively associated with worsened prognosis of HCC. Cao et al. confirmed that glucose‐6‐phosphate dehydrogenase (G6PD) promoted HCC development by downregulating the POR (cytochrome P450 oxidoreductase) pathway, causing enhanced cell proliferation, migration, and invasion and inhibiting ferroptosis [40]. Lu et al. determine that increased G6PD expression contributed to the migration and invasion of HCC cells through inducing epithelial‐mesenchymal transition (EMT) [41]. CDC20 (cell division cycle 20 homolog) functioned as an indicator of poor prognosis in patients diagnosed with HCC [42, 43, 44]. Mechanistically, CDC20 regulated EMT [45] and the Bcl‐2/Bax pathway [46], thus significantly enhancing HCC development and facilitating resistance to radiotherapy. Secreted phosphoprotein 1 (SPP1) acts as a tumor cell growth promoter in HCC, targeted by miR‐181c [47]. TRNP1 (TMF‐regulated nuclear protein 1) was identified to be a promising biomarker for poor HCC prognosis [48]. The oncogenic effects of G6PD, CDC20, SPP1, and TRNP1 displayed in our prognostic model were consistent with those of previous studies.

Additionally, our model demonstrated that CYP2C9, PON1, and ADH4 were negatively associated with poor HCC prognosis, resembling the findings of published studies. CYP2C9 (cytochrome P450 family 2 subfamily C member 9) encodes one of the most abundant and vital drug‐metabolizing enzymes in humans, with its substrate spectrum covering fluoxetine, losartan, phenytoin, tolbutamide, torsemide, S‐warfarin, and numerous nonsteroidal anti‐inflammatory drugs [49]. Dianke Yu reported that the inhibition of CYP2C9 by hsa‐miR‐128‐3p in human hepatocytes resulted in enhanced tumor cell proliferation [50]. PON1 (paraoxonase 1) was negatively related to the vascular invasion status of patients [51]. ADH4 (alcohol dehydrogenase 4), which was significantly reduced in HCC tissues at both transcriptional and translational levels, was confirmed as an independent predictor for OS (HR = 0.154) [52]. Karunakara et al. explored the mechanism underlying the tumor‐suppressive effect of ADH4 and discovered the MEG3/miR664a‐3p/ADH4 axis [53]. To be more concrete, ADH4 was negatively regulated by miR664a‐3p, whose expression was significantly upregulated during HCC. MEG3 (maternally expressed gene 3), an endogenous lncRNA with downregulated expression, functioned as an antagonist for miR664a‐3p [53].

The concept of TMB, which generally stands for the total number of somatic coding mutations in tumors, is based on the premise that an increased number of neoantigen‐generating somatic mutations would make the tumors more responsive to immune checkpoint inhibitors (ICIs), and it is now regarded as a novel biomarker for immunotherapy response prediction [54, 55, 56], despite contradictory opinions that a TMB‐based strategy requires further refining before clinical application [57, 58]. In terms of HCC, whether TMB could play a role in modifying responses to ICIs remains unclear because of very few relevant studies, as reviewed recently by Alessandro Rizzo et al. [59]. Celina Ang et al. conducted comprehensive genomic profiling on 755 samples and reported the median TMB to be only four mutations per megabase, with only six samples (0.8%) having a high TMB [60]. Bufu Tang et al. studied the efficacy of TMB‐related genes as diagnostic and prognostic biomarkers and reported SMG5 and MRPL9 as two significantly associated genes [61]. In this study, we did not find any significant association between TMB and the risk score calculated using our seven‐gene–based model, but it was significantly associated with patient OS. Future studies are needed for further elucidation of the relationship among risk score, TMB, and patient prognosis and whether the combination of our model and TMB serves as an improved strategy for prognosis prediction of patients with HCC.

Our study established a seven‐gene–based model for the estimation of prognosis and immunotherapy responses for patients with HCC. Similarly, previous studies established prognostic models based on various biological characteristics of cancer. For instance, Junyu Long et al. built up a two‐gene–based (EXO1 and TREM1) predictive model by analyzing TP53 expression divergence [62]. Wang et al. assessed immune‐related gene signatures and determined a nine gene–based risk score model (ANGPT1, MAPT, DCK, SEMA3F, IL17D, HSPA4, RBP2, NDRG1, and OSGIN1), which was confirmed to be an independent prognostic indicator after adjusting for other clinical factors [63]. A more recent study revealed the use of a coagulation‐related risk score model for risk stratification and prognosis prediction based on TCGA samples [64]. However, a few studies discussed the efficacy of their model for immunotherapy response prediction. Some studies failed to assess a large number of patient samples, and the lack of cellular and histological experimental results affects the reliability of their prognostic models. Comparatively, our seven‐gene–based model has the following advantages: (1) Our model was established and validated based on four different datasets, namely, TCGA, HCCDB18, GSE14520, and GSE76427. Data of patients' samples obtained from the specialized databases IMvigor210, GSE91061, GSE78220, and GSE135222, especially patients who received immunotherapy, were analyzed. IHC analysis was performed to further strengthen our results. Wide‐ranged data sources have added to the credibility of the results, and our study provides findings that can guide clinical immunological treatment. (2) The metabolism of arachidonic acid is indispensable in biological processes such as inflammation and tumorigenesis, and the study of genes related to this metabolic pathway could provide important information that can guide the treatment of tumors. To the best of our knowledge, this is the first study so far to have focused on the influence of this specific biological process on the prognosis of patients with HCC. (3) In the present study, the risk score was combined with clinicopathological characteristics to further enhance the model's prognostic efficacy. This will further ensure the accuracy of prognostic prediction in clinical practice.

Conclusively, in this work, we determined three distinctive subgroups through data analysis of gene expression related to arachidonic acid metabolism from the TCGA dataset, and we constructed a prognostic seven‐gene–based signature for predicting both prognosis and responses to immune therapy for patients with HCC. The model focused merely on gene expression levels on the mRNA level, with no regard to gene mutations or epigenetic modifications, such as methylation and acetylation. Moreover, the model was validated using the HCCDB18, GSE14520, and GSE76427 databases. Therefore, our seven‐gene–based model was proven to have satisfactory stability and feasibility, which was undoubtedly helpful for clinical application with no extra need for whole‐genome sequencing. Moreover, the use of our model for assessing responses to immune therapy in patients with HCC may have instructive values for guiding clinical medication and individually helping patients find the most suitable treatment strategy. However, there are a few limitations in our current study. First, we did not explore the use of our seven‐gene–based model for HCC diagnosis, mainly because the model has little significance for early recognition of HCC. Second, we briefly described the difference between both risk groups concerning metabolic pathways, but we did not further explore the underlying mechanisms. Third, our study was conducted retrospectively; therefore, the findings are less convincing than those reported by prospective research.

## 5 Conclusion

In this study, we developed a novel prognostic model that incorporates seven genes associated with arachidonic acid metabolism. The model demonstrated satisfactory efficacy in predicting patient prognosis and their responses to immunotherapy in HCC. Although our results did not reveal significant correlations between risk score and TMB, they did suggest that the seven‐gene–based model held promise for predicting immunotherapy outcomes. Moreover, risk score combined with clinical pathological characteristics further improves the prognostic accuracy of our HCC model. Notably, this model also serves as an independent predictive tool in clinical practice.

## Author Contributions

**Xinyu Gu:** conceptualization (equal), data curation (equal), methodology (equal), software (equal), supervision (equal), writing – original draft (lead). **Jing Wang:** formal analysis (equal), investigation (equal), methodology (equal), writing – original draft (supporting). **Jun Guan:** data curation (supporting), methodology (equal), validation (equal), writing – review and editing (equal). **Guojun Li:** funding acquisition (supporting), methodology (equal), visualization (equal). **Xiao Ma:** methodology (equal), software (equal). **Yanli Ren:** visualization (equal), writing – review and editing (supporting). **Shanshan Wu:** writing – review and editing (equal). **Chao Chen:** software (equal). **Haihong Zhu:** conceptualization (equal), funding acquisition (lead), project administration (lead), supervision (equal), writing – review and editing (equal).

## Ethics Statement

The use of TMA containing human tissues was approved by the ethical committee of the Shanghai Outdo Biotech Co. Ltd.

## Consent

The authors have nothing to report.

## Conflicts of Interest

The authors declare no conflicts of interest.

## Supporting information


**Figure S1.** Analysis of the expression of genes related to arachidonic acid metabolism that affected HCC prognosis. (A) Twelve genes involved in arachidonic acid metabolism identified as potential prognostic indicators of HCC OS, nine of which were risk factors and three were protective factors. (B) Six of the 12 genes related to prognosis had significantly upregulated expression in cancer tissues when compared with that in paracancerous normal tissues, while five genes had significantly degraded expression levels. (C) Mutation frequency of 1% for CYP2C8, CYP2C9, GGT7, PTGS1, and CYP4A11, and that of less than 1% for other genes among the 12 genes. (D) Copy number variation of the 12 genes related to prognosis was examined and visualized. HCC: hepatocellular carcinoma; OS: overall survival.

**Figure S2.** Ability of the seven‐gene–based model to predict immunotherapy responses. (A) ssGSEA results showing 40 pathways with significant differences between the high‐ and low‐risk groups. (B‐D) Immune cell infiltration condition in both high‐ and low‐risk groups. A correlation between higher immune scores and higher risks for poor prognosis was observed for both acquired and innate immune responses. (E) ESTIMATE algorithm immune score was positively related to prognostic risk. (F) A higher risk score showing relationship with the expression of genes related to FGFR3, EMT1‐3, base excision repair, nucleotide excision repair, homologous recombination, mismatch repair, DNA replication, DDR, and cell cycle. (G) Positive relationship between TIDE score and prognostic risk. (H) A heatmap showing the association between the expression of each of the seven genes and several immunotherapy‐related features. (I) Results of the pRRophetic algorithm indicating that 65 of the 72 drugs were more effective in treating patients in the high‐risk group, while seven were more effective in treating patients in the low‐risk group. DDR: DNA damage response; EMT: epithelial‐mesenchymal transition; ssGSEA: single‐sample gene set enrichment analysis; TIDE: Tumor Immune Dysfunction and Exclusion.

## Data Availability

All data generated or analyzed during this study are included in this published article [and its supplementary information files].
