# Supplementary figures and images for: Predictive Prognostic Model for Hepatocellular Carcinoma Based on Seven Genes Participating in Arachidonic Acid Metabolism
Source: Cancer Med. 2024 Nov 14;13(22):e70284. doi: 10.1002/cam4.70284 (PMC11561968; doi:10.1002/cam4.70284)

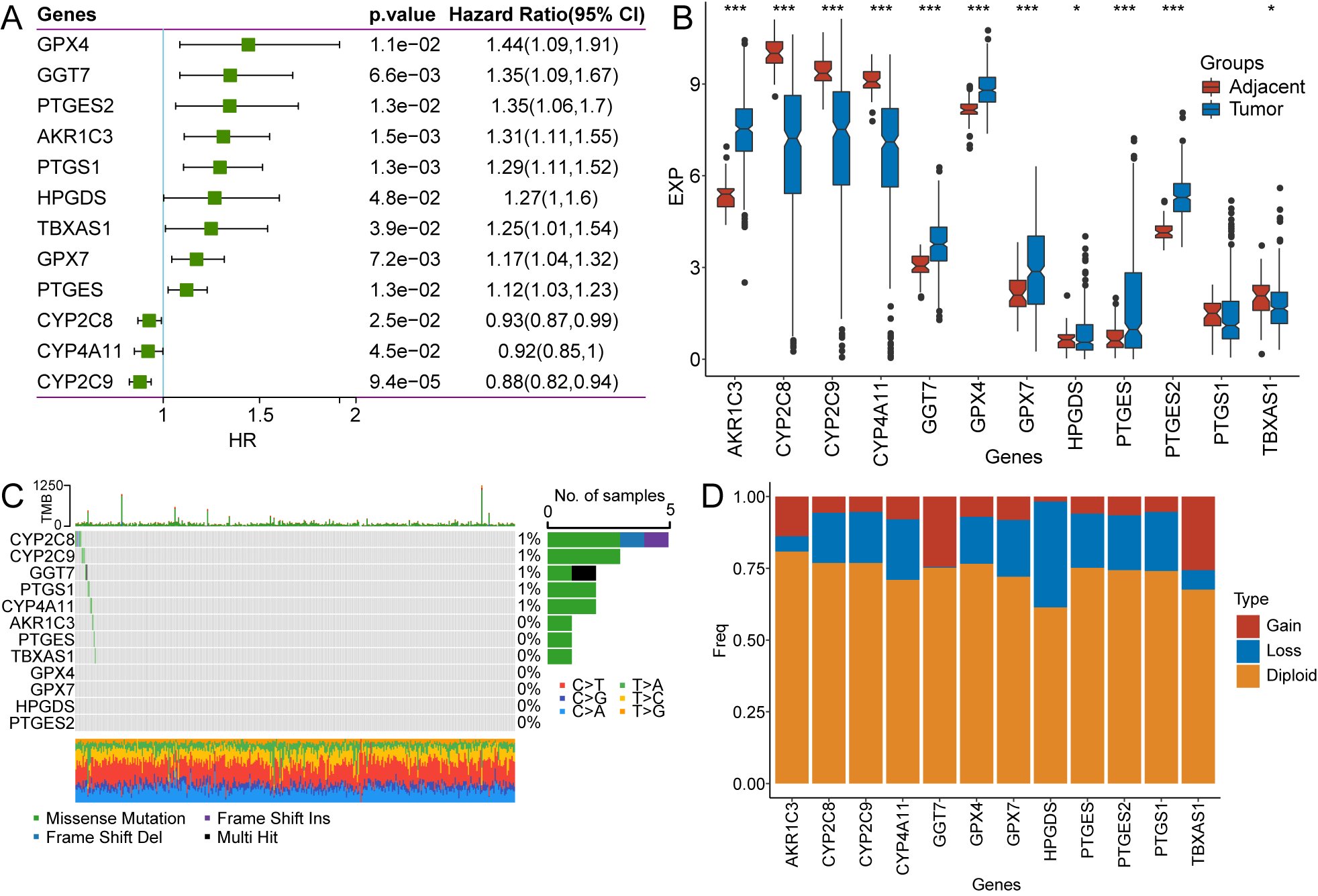

Supplement: Supplementary file 1 — Figure S1. Analysis of the expression of genes related to arachidonic acid metabolism that affected HCC prognosis. (A) Twelve genes involved in arachidonic acid metabolism identified as potential prognostic indicators of HCC OS, nine of which were risk factors and three were protective factors. (B) Six of the 12 genes related to prognosis had significantly upregulated expression in cancer tissues when compared with that in paracancerous normal tissues, while five genes had significantly degraded expression levels. (C) Mutation frequency of 1% for CYP2C8, CYP2C9, GGT7, PTGS1, and CYP4A11, and that of less than 1% for other genes among the 12 genes. (D) Copy number variation of the 12 genes related to prognosis was examined and visualized. HCC: hepatocellular carcinoma; OS: overall survival. [file CAM4-13-e70284-s002.tif]

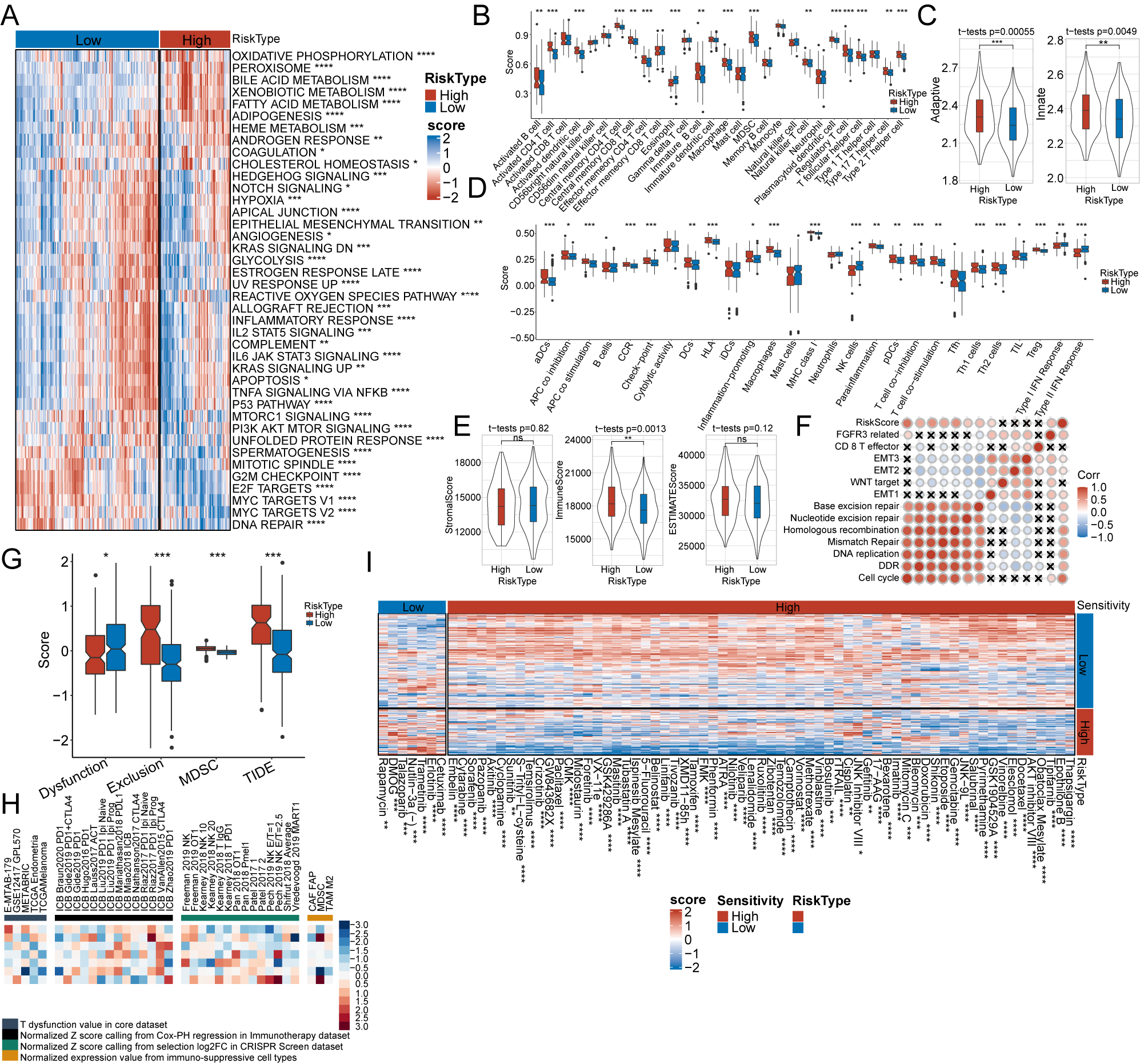

Supplement: Supplementary file 2 — Figure S2. Ability of the seven‐gene–based model to predict immunotherapy responses. (A) ssGSEA results showing 40 pathways with significant differences between the high‐ and low‐risk groups. (B‐D) Immune cell infiltration condition in both high‐ and low‐risk groups. A correlation between higher immune scores and higher risks for poor prognosis was observed for both acquired and innate immune responses. (E) ESTIMATE algorithm immune score was positively related to prognostic risk. (F) A higher risk score showing relationship with the expression of genes related to FGFR3, EMT1‐3, base excision repair, nucleotide excision repair, homologous recombination, mismatch repair, DNA replication, DDR, and cell cycle. (G) Positive relationship between TIDE score and prognostic risk. (H) A heatmap showing the association between the expression of each of the seven genes and several immunotherapy‐related features. (I) Results of the pRRophetic algorithm indicating that 65 of the 72 drugs were more effective in treating patients in the high‐risk group, while seven were more effective in treating patients in the low‐risk group. DDR: DNA damage response; EMT: epithelial‐mesenchymal transition; ssGSEA: single‐sample gene set enrichment analysis; TIDE: Tumor Immune Dysfunction and Exclusion. [file CAM4-13-e70284-s001.tif]
